# Safety and Efficacy of Echo- vs. Fluoroscopy-Guided Pericardiocentesis in Cardiac Tamponade

**DOI:** 10.3390/medicina61020265

**Published:** 2025-02-04

**Authors:** Dejan S. Simeunović, Ivan Milinković, Marija Polovina, Danijela Trifunović Zamaklar, Ivana Veljić, Stefan Zaharijev, Marija Babić, Dejan Nikolić, Valerija Perić, Nina Gatarić, Arsen D. Ristić, Petar M. Seferović

**Affiliations:** 1 Department of Cardiology, University Clinical Center of Serbia, 11000 Belgrade, Serbia; ivan.milinkovic@yahoo.com (I.M.); maki.marijapolovina@gmail.com (M.P.); danijelatrif@gmail.com (D.T.Z.); ivanazivkovic1985@gmail.com (I.V.); szaharijev91@gmail.com (S.Z.); marijabb222@gmail.com (M.B.); valerijaperic125@gmail.com (V.P.); gataricnina@gmail.com (N.G.); arsen.ristic@gmail.com (A.D.R.); 2 Faculty of Medicine, University of Belgrade, 11000 Belgrade, Serbia; denikol27@gmail.com (D.N.); seferovic.petar@gmail.com (P.M.S.); 3 Department of Physical Medicine and Rehabilitation, University Children’s Hospital, 11000 Belgrade, Serbia; 4 Serbian Academy of Sciences and Arts, 11000 Belgrade, Serbia

**Keywords:** cardiac tamponade, pericardiocentesis, fluoroscopy guided, echo guided

## Abstract

*Background and Objectives:* Cardiac tamponade is managed through echo- or fluoroscopy-guided percutaneous pericardiocentesis. The European Society of Cardiology’s Working Group on Myocardial and Pericardial Diseases proposed a triage strategy for these patients. This study evaluated the triage score and compared the safety and efficacy of fluoroscopy- versus echo-guided procedures without additional visualization control. *Materials and Methods:* This prospective observational study included 71 patients with cardiac tamponade from February 2021 to June 2022. Pericardiocentesis was performed using fluoroscopy or echo guidance based on clinical assessment and catheterization laboratory availability, without the additional control of needle/guidewire position or ECG monitoring. Patients were followed for three months. *Results:* The study included 71 patients (52.1% female, mean age 59.7 ± 15.7 years). Malignancy was the most common comorbidity (59.2%). Echo criteria led to urgent procedures in 47.9%, with subcostal access used most often (60.6%), particularly in fluoroscopy-guided procedures (93.8%, *p* = 0.003). The success rate was 97.1%, with minor complications in 14% of patients. Diabetes and malignancy predicted complications regardless of access site or guiding method. The triage score did not affect complication rates or short-term mortality. *Conclusions:* Fluoroscopy- and echo-guided pericardiocentesis without additional visualization control showed no difference in safety or efficacy. Delaying the procedure for patients with a triage score ≥6, or performing it early for those with a low score, did not impact complication rates or mortality, which were more influenced by the progression of the underlying disease.

## 1. Introduction

Cardiac tamponade is a condition that develops due to fluid accumulation in the pericardial space, leading to compressive effects on the myocardium and hemodynamic consequences [1]. The clinical presentation is determined by the pericardial stiffness and the rate of fluid accumulation [2]. A rapid fluid build-up of as little as 200 mL can cause cardiac tamponade, whereas a slow accumulation of up to 2000 mL of pericardial fluid may occur without hemodynamic consequences, depending on myocardial compliance, parietal pericardial thickness, and intracardiac pressures [3]. Cardiac tamponade is treated by percutaneous pericardiocentesis or surgical drainage. Pericardiocentesis is faster, simpler, more comfortable, less invasive, and associated with fewer complications [4]. However, indications for urgent pericardiocentesis can present significant clinical challenges. Small to medium effusions secondary to other treatable conditions (e.g., heart failure or pericarditis) usually do not warrant an urgent procedure. Additionally, large asymptomatic pericardial effusions associated with systemic inflammatory or endocrine diseases can often be treated successfully with medications. In cases of hemodynamic instability or when there is a suspicion of a background disease that can be treated specifically, pericardiocentesis is indicated regardless of the size of the effusion [1,2,3,4,5]. The Working Group on Myocardial and Pericardial Diseases of the European Society of Cardiology (WG on M&P Diseases of ESC) proposed, in 2012, a triage strategy for cardiac tamponade based on available data and expert consensus [4]. This triage score applies to all patients except those in cardiogenic shock, where the procedure has vital indications. It serves as a valuable tool for clinicians in decision-making regarding urgent pericardial drainage by integrating data on etiology, clinical presentation, and imaging. This minimizes the possibility of over- or underestimating urgency, thereby reducing the risk of deferring the procedure in indicated cases or causing complications in cases where it could have been postponed. However, this triage score has not been evaluated in a prospective study. According to the 2004 Guidelines on the Diagnosis and Management of Pericardial Diseases, blind pericardiocentesis is contraindicated due to the high risk of complications [1,4]. Echo-guided and fluoroscopy-guided procedures are considered equally safe [6,7]. Based on the available data, only two studies have compared these two methods [8,9]. However, in both trials, the echo-guided procedure was supplemented by the additional control of the puncture needle/guidewire position using fluoroscopy or agitated saline solution. This distinction is important because echo-guided procedures without additional control methods are more widely available and can simplify patient management.

The primary aims of this study are to evaluate, in a real-life population, the triage score for cardiac tamponade proposed by the WG on M&P Diseases of ESC in 2012. Additionally, the safety and efficacy of echo-guided versus fluoroscopy-guided procedures without additional visualization control will be compared in patients with cardiac tamponade.

## 2. Methods

The prospective observational study included consecutive patients hospitalized or referred to the University Clinical Center of Serbia for pericardiocentesis between February 2021 and June 2022. All patients had detailed information collected, including their previous medical history, physical examination findings with vital parameters, echocardiography, chest X-rays, and routine laboratory analyses (Supplementary Table S1). Echocardiographic measurements of pericardial effusion were taken in the supine position during diastole. Based on the maximum measurements in standard echocardiographic projections, effusions were semi-quantitatively categorized as small (<10 mm), medium (10–20 mm), or large (>20 mm). Echocardiographic criteria for tamponade were defined as the presence of diastolic ventricular or atrial collapse (not merely the size of the effusion). Clinical criteria involved the severity of the clinical presentation, and hemodynamic criteria included systolic blood pressure below 90 mmHg. According to official guidelines [1], the clinical assessment of the physician, and the availability of the catheterization laboratory, pericardiocentesis was performed using either an echo-guided or fluoroscopy-guided method (details of the procedures are described in references [1,5]). In the echo-guided procedure, after the initial marking of the access site, the procedure proceeded with clinical monitoring of the patient but without the additional control of the puncture needle/guidewire position or ECG monitoring. Control echocardiography was performed only in cases of suspected malposition of the drainage catheter or worsening of the patient’s general condition. Pericardial fluid was analyzed for biochemical, bacteriological, cytological, immunocytochemical, Koch’s bacillus, and Lowenstein characteristics. The duration, amount of fluid drained, and early and late complications of the procedure were recorded. Additionally, all other diagnostic methods performed were documented. A complete echocardiographic examination was conducted before discharge. The triage score proposed by the WG on M&P Diseases of the ESC [4] was calculated retrospectively to assess its value in determining the urgency for pericardiocentesis. Furthermore, patients were followed up for in-hospital and three-month mortality. As outcome measures, we analyzed the procedural success rate, defined as the success or number of attempts to perform the procedure, as well as all complication rates, including periprocedural mortality. The methodology described above did not require approval from the institutional ethics committee, as the decision to perform echo- or fluoroscopy-guided pericardiocentesis was based on the availability of the catheterization laboratory or the urgency of the procedure.

### Statistical Analysis

Continuous variables were presented as mean values with standard deviation, and the *t*-test was used for statistical analysis. The Mann–Whitney U test was applied to variables assumed not to follow a normal distribution. Categorical variables were presented as percentages, and the Chi-square test was used to assess between-group differences. Fisher’s exact test was applied for small sample sizes. Multivariate Cox regression analysis was performed to identify predictors of complications. For complications and survival analysis, Kaplan–Meier curves were constructed, and the log-rank test was used for comparisons. A *p*-value of <0.05 was considered statistically significant. All statistical analyses were performed using SAS version 9.4 (SAS Institute, Inc., Cary, NC, USA).

## 3. Results

This prospective study included 71 consecutive patients with cardiac tamponade, with a mean age of 59.7 ± 15.7 years, of whom 52.1% were female. The most common comorbidity (or pericardial effusion etiology) at presentation was malignant disease (59.2%), primarily lung carcinoma (29.5%), breast carcinoma (11.3%), and lymphoproliferative disease (8.5%). The predominant cardiovascular risk factors were arterial hypertension (46.5%) and smoking (32.4%). In only three cases, a cardiovascular intervention preceded the occurrence of pericardial effusion (coronary bypass surgery with mitral valve repair, pacemaker implantation, and device explantation). Chronic pericardial effusion and systemic autoimmune disease were significantly more frequent in the group with fluoroscopy guidance (*p* = 0.007 and *p* = 0.001, respectively), while diabetes mellitus was significantly more frequent in the group with echocardiography guidance (*p* = 0.026) (Table 1).

The predominant symptom at presentation was dyspnea (63.4%), followed by chest pain (15.5%) and orthopnea (11.3%). Less frequent symptoms included cough (4.2%), arrhythmias, and vertigo (1.4% each), while two patients were completely asymptomatic (2.8%). The rapid worsening of symptoms was reported by 21 patients (29.6%). On physical examination, prominent neck veins were detected in 8 patients (11.2%), chest rales in 13 patients (18.3%), obstructive breathing in 7 patients (9.8%), an enlarged liver in 6 patients (8.4%), and peripheral edema in 20 patients (28.2%). The echocardiography-guided group had higher C-reactive protein values compared to the fluoroscopy-guided group (*p* = 0.036) (Table 2).

Low voltage was the most frequent ECG finding (39.4%), followed by tachycardia (heart rate ≥110/min) in 15.5% of cases and electrical alternans (16.9%). Concave ST segment elevation was registered in only four cases (5.6%).

In determining the need for urgent pericardiocentesis, echo criteria were crucial in 34 patients (47.9%), clinical criteria in 23 patients (32.4%), and hemodynamic criteria in 6 cases (8.4%), while 8 patients (11.3%) had none of the aforementioned criteria.

Pericardial effusion >2 cm was detected in 81.7% of patients, while others had a maximal effusion ranging from 1 to 2 cm. The majority of patients had circumferential pericardial effusion (92.9%), while others had asymmetrical or loculated distributions. Right atrial collapse was the most frequent echo sign of hemodynamic significance (67.6%), followed by right ventricular collapse (64.8%), “swinging heart” (30.9%), and dilated inferior vena cava (16.9%). The least frequent sign was paradoxical interventricular septal movement (“septal bounce”) (5.6%).

Pleural effusion accompanied pericardial effusion in 66.2% of patients, of which 24% had pleurocentesis performed before or after pericardiocentesis.

The predominant access site was subcostal (60.6%), which was more common in fluoroscopy-guided procedures (93.8%, *p* = 0.003). In contrast, in echo-guided procedures, the apical intercostal and subcostal approaches were almost equally represented (~49% and ~51%, respectively).

Pericardiocentesis was successful in 97.1% of cases. The procedure failed in asymmetrical effusions, primarily localized behind the posterior ventricular wall. More than one attempt was needed in six cases (8.4%). Minor complications were detected in 10 patients (14%), most frequently pleural puncture (5.6%) in the apical intercostal approach and pneumothorax (4.2%) regardless of the access site. There were no major complications requiring surgery.

A hemorrhagic effusion was evacuated in most cases (62%). The average initial drainage was 740 ± 406.76 mL, the mean total drainage was 1550 ± 971.32 mL, with an average drainage duration of 4 days (Table 3).

In eight cases (11.3%), there was a conflict between direct microscopy, which identified malignant cells, and immunohistochemistry, which did not confirm the presence of these cells. However, malignant disease was confirmed in six patients (8.4%) after additional imaging. Tuberculosis was not identified, and none of the patients had purulent pericarditis, although eight samples of pericardial fluid (11.3%) contained microorganisms classified as contamination.

During the three-month follow-up, the mortality rate was 15.5% in the entire population, with eight cases (72%) attributed to malignant disease. There were no serious events related to the pericardiocentesis procedures. Multifactorial analysis showed that diabetes mellitus (*p* = 0.032) and malignant disease (*p* = 0.024) were predictors of complications (in this study, pleural puncture/pneumothorax), regardless of the access site or guiding method (Table 4).

**Figure 1:**
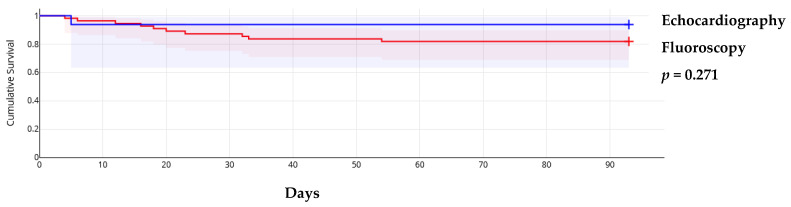
Kaplan–Meier survival curves according to procedure guiding method.

**Figure 2:**
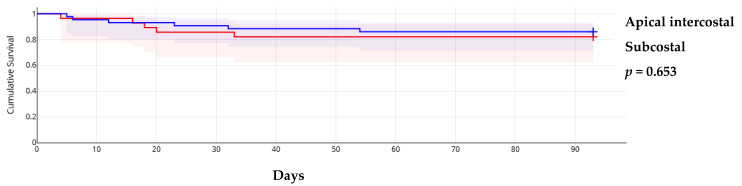
Kaplan–Meier survival curves according to approach site.

The triage score from the WG on M&P diseases of ESC showed that in 23 cases (32.4%), pericardiocentesis was postponed despite patients having a score ≥6, while in 7 cases (9.9%), the procedure was performed early in those with a low score. In the remaining cases, the assessment was based on the score (Supplementary Table S2). This approach did not influence short-term mortality, which was primarily determined by the progression of the primary, usually malignant, disease. Additionally, early procedures did not result in significantly more minor complications (3 out of 10 registered minor complications). Neither the guiding method nor the approach site influenced overall survival (Figure 1 and Figure 2).

## 4. Discussion

The current prospective trial did not show a difference in safety and efficacy between fluoroscopy- and echo-guided procedures without additional visualization control. The overall success rate was high (97.1%), with a low complication rate. Furthermore, pericardiocentesis performed in patients whose procedure could be postponed according to the triage score of WG on M&P diseases of ESC did not lead to significantly higher complications. Early and postponed pericardiocentesis did not influence short-term mortality, which was more dependent on the progression of the primary, usually malignant, disease. Malignant disease is the most common background condition in cardiac tamponade patients (59.2%), with lung carcinoma, breast carcinoma, and lymphoproliferative disease being the most prevalent. Malignant disease, along with diabetes, is a predictor of complications after pericardiocentesis, regardless of approach site or guiding method.

To the best of our knowledge, this is the only available study comparing the safety and efficacy between fluoroscopy- and echo-guided procedures without additional visualization control during the procedure. In a similar trial by Kim et al. [8], the correct position of the guide wire was verified under fluoroscopy, while in the study by Cheong et al. [9], needle tip position was occasionally controlled with echo or agitated saline. Since blind pericardiocentesis was declared contraindicated, the success rate of pericardiocentesis rose to around 99%, with a lower complication rate of 1–3% and low mortality [8,9,10,11,12]. Although our trial showed a somewhat lower success rate (97%), no additional visualization method to assess needle/guide wire position was used in the echo-guided group. This could explain the slightly higher minor complication rate (14%), such as pleural puncture (5.6%) and pneumothorax (4.2%). However, no major complications requiring surgical intervention were recorded, unlike other large series of procedures [8,9,10,11]. It is possible that the high procedure volume at the department where this trial was conducted influenced the lower occurrence of major complications. Additionally, using agitated saline to control needle position may prolong the switching process between the needle and guide wire, potentially increasing the risk of major complications.

The etiology of pericardial effusion varies between high- and low-income countries [13,14,15,16,17,18]. In high-income economies, iatrogenic complications and malignant diseases are the predominant causes, while in low-income countries, infective agents such as HIV and tuberculosis are more frequent [13]. Pericardial effusions in malignant disease are expected to rise, not only due to the increasing prevalence of malignancy but also because contemporary treatments prolong survival and cause effusions as side effects [14]. In cardiac tamponade, malignant disease accounts for 20–80% of cases, depending on the country’s development status and the predominant pathology in the center where the trial is conducted. Developed countries and tertiary institutions with a high volume of cardiovascular surgical procedures tend to have lower rates of malignant pericardial effusions (42–48%) [9,10,11,12,15]. In our study, 59.2% of patients had an accompanying malignant disease, with lung carcinoma being the most common (29.5%), followed by breast carcinoma (11.3%) and lymphoproliferative disease (8.5%). The higher prevalence of breast carcinoma and lymphoproliferative disease may suggest that, besides lifestyle, other factors such as stress, pollution, and the consequences of recent military conflicts may influence the general population.

Orthopnea and dyspnea are valuable first triage criteria when evaluating patients with cardiac tamponade. These symptoms are assigned three points on the triage score of WG on M&P diseases of ESC, with an additional two points for the rapid worsening of symptoms. In our trial, orthopnea/dyspnea were present in 74.7% of cases, which aligns with major case studies (51–89%) [9,11,12,16]. The rapid worsening of symptoms was noted in 29.6% of cases.

Echocardiographic signs of cardiac tamponade were present in 47.9% of our patients, consistent with findings in the literature (46–53%) [12,13,14,16,17,18,19,20,21,22,23]. Circumferential effusion was the most common (92.9%), with a size >2 cm in 81.7% of cases. This is also assigned three points on the triage score of WG on M&P diseases of ESC.

Access site selection reflects local practices, regardless of the guiding method. Among centers performing echo-guided procedures, the rate of subcostal approach varies between 48 and 83% [10,22], while subcostal approach is more common in fluoroscopy-guided procedures (up to 93%) [23], due to better visualization of the halo phenomenon. In our trial, the predominant access site was subcostal (60.6%), more frequent in fluoroscopy-guided procedures (93.8%, *p* = 0.003). In echo-guided procedures, the apical intercostal and subcostal approaches were almost equally represented (~49% vs. ~51%). This is consistent with the approach at the Mayo Clinic, where preference is given to the access site closest to the effusion, limiting injury to vital structures like the liver, heart, and coronary vessels, which is often the apical approach [4,6].

To avoid hemodynamic consequences from rapid fluid evacuation or pericardial decompression syndrome, it is generally recommended that initial drainage does not exceed 1000 mL [4,6]. In our trial, the average initial drainage volume was 740 ± 406.76 mL, with a mean drainage duration of 4 days, in line with the recommendation to limit drainage duration to avoid potentially fatal infectious complications [1,2,4,6].

Routine biochemical analysis of pericardial effusion has limited sensitivity and specificity, as most effusions are exudates [15,24,25]. Cytology has a sensitivity of around 44% (67–92%) [25,26,27] and a specificity up to 100% [26,27,28]. In our trial, the sensitivity for diagnosing new malignant diseases was 38%, while a definitive diagnosis often required additional imaging methods. The early detection of malignant cells in pericardial effusion can significantly impact diagnosis, treatment, prognosis, and outcomes [29,30]. The WG on M&P diseases of ESC also recognizes the need for pericardiocentesis when specific conditions such as malignancy or infection are suspected.

During the 3-month short-term follow-up, the majority of patients who died (15%) had malignant disease. This is consistent with available data, where mortality is around 15% and is associated with the etiology of the effusion and other comorbidities [31]. The guiding method and approach site did not influence the outcomes. Additionally, malignant disease and diabetes mellitus were independent predictors of procedural complications, regardless of the approach site or guiding method. This could be an accidental finding, although patients with malignant disease, particularly pulmonary conditions, may be more susceptible to complications, and diabetes may exacerbate the progression of pulmonary disease, leading to more procedural adverse events [32].

A comparison of the triage score for cardiac tamponade of the WG on M&P diseases of ESC from 2012 and urgency assessments made without using the score showed that in 32% of cases, the procedure was postponed even when urgency criteria were met. This did not lead to higher short-term mortality. On the other hand, in 10% of cases, the score did not indicate urgency, yet the procedure was performed early, without an increase in complications. This suggests that patients with a triage score ≥6 can sometimes be safely postponed, which is important to avoid the complications of emergency procedures in centers that may not be sufficiently equipped or trained.

## 5. Limitations

The major limitations of this study include the relatively small population, non-randomized approach, and short follow-up period. The study was conducted in a specialized tertiary institution with a large volume of pericardiocentesis procedures, where patients are referred from other departments, particularly oncology units, and after periprocedural complications. This limits the generalizability of the conclusions. Due to the heterogeneous nature of the medical records, some parameters could not be analyzed. Additionally, it is possible that some procedures were not included in the final analysis because they were performed in other units of the University Clinical Center.

## 6. Conclusions

The expansion of invasive and interventional treatment methods for cardiovascular diseases, as well as for patients with malignant diseases, will increase the need for pericardiocentesis in the future. Malignant disease is also a predictor of shorter survival and procedural complications. Pericardiocentesis guided by echo or fluoroscopy has become the standard treatment for cardiac tamponade. Since fluoroscopy is not widely available, the echo-guided procedure is a fast, simpler, and efficient method that can be conducted safely without additional control of needle/guide wire position after access to the pericardial space. This is particularly true in high-volume centers, as the complication rate is directly proportional to operator experience and the center’s procedural volume. This highlights the importance of proper education in all units, from primary to tertiary levels. However, both modalities are equally useful for a safe procedure and in an imminently life-threatening situation a bed-side, echo-guided pericardiocentesis could be considered, but this is the exception not the rule. The triage score for cardiac tamponade from the WG on M&P Diseases of ESC, established in 2012, is a useful tool for clinical decision-making. However, a comprehensive and individualized approach to each patient may offer an advantage in triage. Definitive results can be obtained from larger, randomized clinical trials with longer follow-up.

## Figures and Tables

**Table 1:** Demography, clinical characteristics, and comorbidities.

Tested Parameters	All (*n* = 71)	Echocardiography Guide (*n* = 55)	Fluoroscopy Guide (*n* = 16)	*p*-Value *
Demography				
Gender, female, *n* (%)	37 (52.1)	26 (47.3)	11 (68.8)	0.162
Age, years, mean (SD)	59.7 (15.7)	60.33 (16.3)	57.3 (13.4)	0.676
Clinical characteristics				
Systolic pressure, mmHg, mean (SD)	120.3 (21.0)	120.3 (20.4)	120.31 (23.1)	0.528
Diastolic pressure, mmHg, mean (SD)	76.2 (12.3)	76.1 (12.75)	76.50 (11.57)	0.872
Heart rate, per min, mean (SD)	95.9 (16.2)	96.6 (16.7)	93.2 (14.6)	0.962
Tamponade, *n* (%)	63 (88.7)	49 (89.1)	14 (87.5)	0.787
Comorbidities				
Hypertension, *n* (%)	33 (46.5)	27 (49.1)	6 (37.5)	0.413
Diabetes mellitus, *n* (%)	12 (16.9)	12 (21.8)	0	0.026
Coronary disease, *n* (%)	4 (5.6)	4 (7.3)	0	0.568
Atrial fibrillation, *n* (%)	12 (16.9)	11 (20.0)	1 (6.3)	0.275
CVI/TIA, *n* (%)	5 (7.0)	5 (9.1)	0	0.425
Chronic pericardial effusion, *n* (%)	10 (14.1)	4 (7.3)	6 (37.5)	0.007
COPD, *n* (%)	3 (4.2)	1 (1.8)	2 (12.5)	0.125
Chronic kidney disease, *n* (%)	4 (5.6)			
Hypothyroidism, *n* (%)	3 (4.2)	1 (1.8)	2 (12.5)	0.125
Systemic autoimmune disease, *n* (%)	3 (4.2)	0	3 (18.8)	0.001
Malignant disease before pericardiocentesis, *n* (%)	24 (33.8)	18 (32.7)	6 (37.5)	0.722
Malignant disease total, *n* (%)	42 (59.2)	33 (60.0)	9 (56.3)	0.788
Recent infection, *n* (%)	12 (16.9)	9 (16.4)	9 (18.8)	0.823
Obesity, *n* (%)	6 (8.5)	6 (10.9)	0	0.326
Smoking, *n* (%)	23 (32.4)	21 (38.2)	2 (12.5)	0.053

CVI—cerebrovascular insult; COPD—chronic obstructive pulmonary disease; SD—standard deviation; TIA—transitory ischemic attack; * *p*-value was calculated using *t*-test for all continuous variables; Chi-square test was used for categorical variables except for coronary disease, COPD, chronic kidney disease, and hypotyreosis systemic autoimmune disease, where Fisher’s exact test was used.

**Table 2:** Laboratory values.

Tested Parameters	All (*n* = 71)	Echocardiography Guide (*n* = 55)	Fluoroscopy Guide (*n* = 16)	*p*-Value *
Leukocyte, 10^9^/L, median (min–max)	8.1 (5.5–10.9)	10.0 (6.5)	6.9 (2.8)	0.096
Hemoglobin, g/L, mean (SD)	119.0 (21.9)	117.5 (21.9)	124.2 (16.7)	0.225
Thrombocyte, 10^9^/L, median (min–max)	260 (175–354)	259 (176–354)	276 (156–356)	0.936
CRP, mg/L, median (min–max)	35.4 (7.6–81.9)	41.9 (10.7–85.2)	8.6 (2.6–42.6)	0.036
Glycaemia, mmol/L, mean (SD)	6.1 (2.3)	6.2 (2.3)	5.8 (2.1)	0.321
Na^+^, mmol/l, mean (SD)	138.2 (4.9)	138.2 (4.7)	138.3 (5.7)	0.774
K^+^, mmol/l, mean (SD)	4.4 (0.6)	4.45 (0.6)	4.1 (0.3)	0.074
Hs troponin-T, ng/L, median (min–max)	14 (9–26)	16 (9–29)	9 (7.5–15.7)	0.152
d-dimer, mg/L FEU, median (min–max)	3.7 (1.3–9.3)	3.7 (1.4–9.6)	2.2 (0.5–8.8)	0.463
BNP, pg/mL, mean (SD)	131 (57–249)	157 (57–268)	125 (36–142)	0.239
INR, median (min–max)	1.1 (0.9–1.2)	1.1 (0.9–1.2)	1.1 (0.9–1.6)	0.695
eGFR < 60, mL/min/1.73 m^2^, mean (SD)	22 (31.0)	19 (34.5)	3 (18.8)	0.358

BNP—brain natriuretic peptide; CRP—C reactive protein; eGFR—estimated glomerular filtration rate; INR—international normalized ratio; SD—standard deviation; * *p*-value was calculated using *t*-test.

**Table 3:** General information on pericardiocentesis.

Parameters	All (*n* = 71)	Echocardiography Guide (*n* = 55)	Fluoroscopy Guide (*n* = 16)	*p*-Value *
Access site, *n* (%) ▪ Apical intercostal ▪ Subcostal	28 (39.4) 43 (60.6)	27 (49.1) 28 (50.9)	1 (6.3) 15 (93.8)	0.003
Complications	15 (21.1)	12 (21.8)	3 (18.8)	0.548
Macroscopic characteristics of pericardial effusion, *n* (%) ▪ Hemorrhagic ▪ Turbid ▪ Serohemorrhagic ▪ Serous	44 (62.0) 2 (2.8) 6 (8.5) 18 (6.8)	37 (67.3) 1 (1.8) 4 (7.3) 13 (23.6)	7 (43.8) 2 (12.5) 2 (12.5) 6 (37.5)	0.209
Microscopic characteristics of pericardial effusion, *n* (%) ▪ Exudate ▪ Chylopericardium ▪ Malignant cells ▪ Transudate	42 (59.2) 2 (2.8) 25 (35.2) 2 (2.8)	32 (58.2) 1 (1.8) 20 (36.4) 2 (2.6)	10 (62.5) 1 (6.3) 5 (31.3) 0	0.759
Initial drainage volume, ml, median, (min–max)	740 (625–975)	745 (537–1000)	650 (450–950)	0.580
Drainage duration, days, median, (min–max)	4 (3–6)	4 (3–6)	4 (3–6.5)	0.822

* *p*-value was calculated using Mann–Whitney U test for continuous variables; Chi-square test was used for categorical variables.

**Table 4:** Predictors of complications after pericardiocentesis.

Parameters	HR	95% CI	*p*-Value
Female gender	1.781	0.276–11.495	0.544
Age	0.992	0.918–1.071	0.830
Fluroscopic control	0.572	0.039–8.360	0.683
Diabetes mellitus	0.091	0.010–0.813	0.032
Hypertension	0.123	0.014–1.041	0.054
Smoking	0.590	0.081–4.308	0.603
Obesity	0.168	0.009–3.129	0.232
Atrial fibrillation	1.127	0.115–11.013	0.918
Chronic kidney disease	0.869	0.031–24.215	0.934
**Malignant disease**	0.065	0.006–0.703	0.024

## Data Availability

Data supporting the obtained results can be obtained from the first author upon reasonable request.

## Supplementary Materials

The following supporting information can be downloaded at: https://www.mdpi.com/article/10.3390/medicina61020265/s1, Table S1: List of study parameters; Table S2: Comparison of triage score and clinical decision for urgent pericardiocentesis.
