# Supplementary material for: Safety and Efficacy of Echo- vs. Fluoroscopy-Guided Pericardiocentesis in Cardiac Tamponade
Source: Medicina (Kaunas). 2025 Feb 4;61(2):265. doi: 10.3390/medicina61020265 (PMC11857578; doi:10.3390/medicina61020265)
Supplement: Supplementary file 1 [file medicina-61-00265-s001.zip › medicina-3373701-supplementary.pdf]

Supplementary Table S1. List of study parameters.

1. General Information

- Gender (male, female)
- Age (years)
- Method of procedure control (fluoroscopy, echocardiography)
- Date of beginning of the disease
- Date of procedure
- Delay of the procedure (yes, no)
- Access point for the pericardiocentesis (apical, subcostal)
- Procedure Success/Complications (yes, no)
- Amount of drained effusion during puncture (ml)
- Amount of drained effusion total (ml)
- Macroscopic appearance of pericardial effusion (serous, sero-emorrhagic, hemorrhagic, opalescent)
- Length of drainage (days)
- Microscopic finding of pericardial effusion (exudate, transudate, malignant cells, chylopericardium, purulent, tuberculous)
- Immunocytochemistry of pericardial effusion
- Bacteriology/BK pericardial effusion
- Dominant symptom (dyspnea, orthopnea, palpitations, vertigo, hypotension, shock, oliguria/anuria, chest pain, cough, asymptomatic)
- Rapid worsening of symptoms (yes, no)
- Tamponade (clinical, echo or hemodynamic criteria, no tamponade)
- Vital status at the end of follow up period (dead, alive)
- Suspected disease/Final diagnosis

2. Co-morbidities

- Diabetes mellitus
- Hypertension
- Hyperlipidemia
- Smoker (active/former)
- Obesity
- Coronary artery disease/Peripheral vascular disease
- Aortic dissection
- Heart failure
- Congenital heart disease/valvular disease
- Atrial fibrillation
- Renal failure
- Stroke
- Chronic obstructive pulmonary disease
- Myocarditis
- Hypothyroidism
- Hyperthyroidism
- Systemic autoimmune disease
- Malignancy/Radiation therapy
- Deep venous thrombosis
- Tuberculosis
- Pacemaker implantation
- Liver cirrhosis

- Depression
- Previous pericardial effusion (chronic)
- Previous pleural punction
- Immunodeficiency/immunosuppression
- Previous pulmonary embolism
- Recent infection/fever
- Recent heart attack
- Recent trauma
- Recent procedure/operation
- Other

3. Physical examination parameters

- Prominent jugular veins (yes, no)
- Lungs rales/obstruction (yes, no)
- Pericardial friction (yes, no)
- Ascites (yes, no)
- Enlarged liver (yes, no)
- Peripheral oedema (yes, no)
- Systolic blood pressure (mmHg)
- Diastolic blood pressure (mmHg)
- Heart rate (per min)
- Pulsus paradoxus (yes, no)
- Pulse oxygen saturation (%)

4. Laboratory parameters

- Leucocytes (10^9/L)
- Hemoglobin (g/l)
- Platelets (10^9/L)
- CRP - C-reactive protein (mg/L)
- Glycemia (mmol/L)
- eGFR - Estimated glomerular filtration rate (mL/min/1,73 m2)
- Sodium (mmol/)
- Potassium (mmol/)
- AST - Aspartate aminotransferase (U/L)
- ALT - Alanine aminotransferase (U/L)
- *LDH - Lactate dehydrogenase* (U/L)
- CK-Creatine kinase (U/L)
- High sensitive Troponin T (ng/L)
- INR-International normalized ratio
- d-dimer (mg/L FEU)
- BNP-Brain natriuretic peptide (pg/mL)
- Ntpro BNP -N-Terminal Pro–B-Type Natriuretic Peptide (pg/mL)

5. Electrocardiography parameters

- Tachycardia (yes, no)
- Microvoltage (yes, no)
- Electrical alternans (yes, no)
- PR segment depression (yes, no)
- ST segment elevation (yes, no)
- Other

6 Echocardiography parameters

- Maximal measured diameter of pericardial effusion in diastole (cm)
- Size Category (less than 1cm, 1-2cm, more than 2cm)
- Diameter of pericardial effusion in diastole in subcostal projection (cm)
- Diameter of pericardial effusion in diastole in apical 4 chamber projection (cm)
- Distribution of pericardial effusion (asymmetrical/localized, circumferential)
- Right atrial collapse (>from 1/3 of cardiac cycle)
- Right ventricular collapse (yes, no)
- Left atrial collapse (yes, no)
- Dilated inferior vena cava above 2.5cm/inspiratory collapse less than 50%
- Heart palpitations
- Septal bounce/signs of constriction (yes, no)
- Left ventricular ejection fraction (%)
- Left ventricular hypertrophy (yes, no)

7. Chest X ray parameters

- Cardiomegaly (yes, no)
- Lung disorder (yes, no)
- Pleural effusion (yes, no)

8. Additional diagnostic procedures

- Chest computerized tomography
- Abdominal echo
- Gynecological examination
- Neck/soft tissue echo
- Cardiac nuclear magnetic resonance heart
- Heart catheterization
- Thyroid hormones
- Tumour markers
- Immunological analyses

Supplementary Table S2. Comparison of triage score and clinical decision for urgent pericardiocentesis

Patient No	Age, Year (gender)	Total Score	Pericardiocentesis Could Have Been Postponed	Pericardiocentesis Performed Later	Pericardiocentesis Performed According to Score	Vital Status (deceased)	Complications
1	93 (F)	11,5			x	x	Passive air suction though drain
2	48 (F)	9,5		x			
3	74 (F)	3,0	x				
4	46 (M)	10,0			x		
5	36 (M)	8,0		x			
6	72 (M)	7,5		x			
7	73 (M)	6,0		x			Pleural puncture
8	78 (F)	5,0			x		
9	63 (M)	4,0			x		Small pneumothorax
10	49 (M)	15,5			x		
11	65 (F)	6,5		x			
12	56 (M)	6,0		x			
13	53 (F)	14,0			x		
14	68 (F)	11,5		x			
15	54 (M)	8,5		x		x	
16	55 (F)	12,0			x		
17	77 (F)	5,5			x		
18	76 (M)	3,5	x				Pneumopericardium
19	67 (M)	7,5			x		
20	84 (F)	7,0			x		
21	64 (M)	4,0	x				
22	42 (F)	8,0			x		
23	21 (M)	8,5		x			
24	64 (F)	10,5					Gudewire break
25	64 (M)	9,0		x		x	Pleural punction
26	79 (F)	7,0			x	x	
27	51 (F)	6,0			x		
28	50 (M)	2,0	x				Small pneumopericardium
29	73 (M)	8,5			x	x	
30	73 (F)	10,5			x	x	
31	57 (F)	8,5			x		
32	56 (F)	6,0		x			Pleural punction

Patient No	Age, Year (gender)	Total Score	Pericardiocentesis Could Have Been Postponed	Pericardiocentesis Performed Later	Pericardiocentesis Performed According to Score	Vital Status (deceased)	Complications
33	32 (F)	5,0	x				
34	59 (F)	7,5			x	x	
35	71 (M)	9,0			x	x	
36	44 (F)	5,0			x		
37	32 (M)	6,5					
38	50 (F)	8,0		x			
39	75 (M)	7,5		x			
40	62 (M)	12,5			x		
41	74 (F)	6,5		x			
42	66 (F)	13,5		x			Simulataneous pleural and pericardial puncture
43	63 (M)	11,5			x	x	
44	24 (F)	3,0	x				
45	81 (M)	6,0			x		
46	61 (F)	10,0			x		
47	60 (M)	10,5			x		
48	57 (M)	7,5			x		
49	74 (M)	5,0			x		
50	56 (M)	7,5		x			
51	28 (M)	7,5		x		x	
52	69 (F)	7,0					
53	51 (F)	14,5		x			
54	51 (F)	10,0				x	
55	68 (M)	9,5		x			
56	56 (M)	10,5			x		
57	71 (M)	6,5			x		
58	56 (F)	8,5			x		
59	88 (F)	6,5			x		
60	39 (M)	6,0		x			
61	64 (M)	5,5	x				Small pneumothorax and subcutaneous emphysema
62	25 (F)	4,5			x		
63	55 (F)	10,5			x		

Patient No	Age, Year (gender)	Total Score	Pericardiocentesis Could Have Been Postponed	Pericardiocentesis Performed Later	Pericardiocentesis Performed According to Score	Vital Status (deceased)	Complications
64	65 (F)	10,5			x		
65	67 (M)	8,5			x		
66	81 (M)	9,0		x			
67	62 (F)	6,0		x			
68	51 (F)	6,0		x			
69	31 (F)	7,5			x		
70	73 (M)	7,5			x		
71	63 (F)	3,0			x		

Green—pericardiocentesis performed without postponing, according to score (match with score); Red—pericardiocentesis postponed, but had to be performed after admission according to score; Yellow—pericardiocentesis performed, but could have been postponed according to score.
